# 4D Printing of Body Temperature-Responsive Hydrogels Based on Poly(acrylic acid) with Shape-Memory and Self-Healing Abilities

**DOI:** 10.1021/acsabm.2c00939

**Published:** 2023-01-26

**Authors:** Turdimuhammad Abdullah, Oguz Okay

**Affiliations:** Department of Chemistry, Istanbul Technical University, 34469Maslak, IstanbulTurkey

**Keywords:** 4D printing, Shape-memory hydrogels, Self-healing, Body temperature, Polyacrylic acid

## Abstract

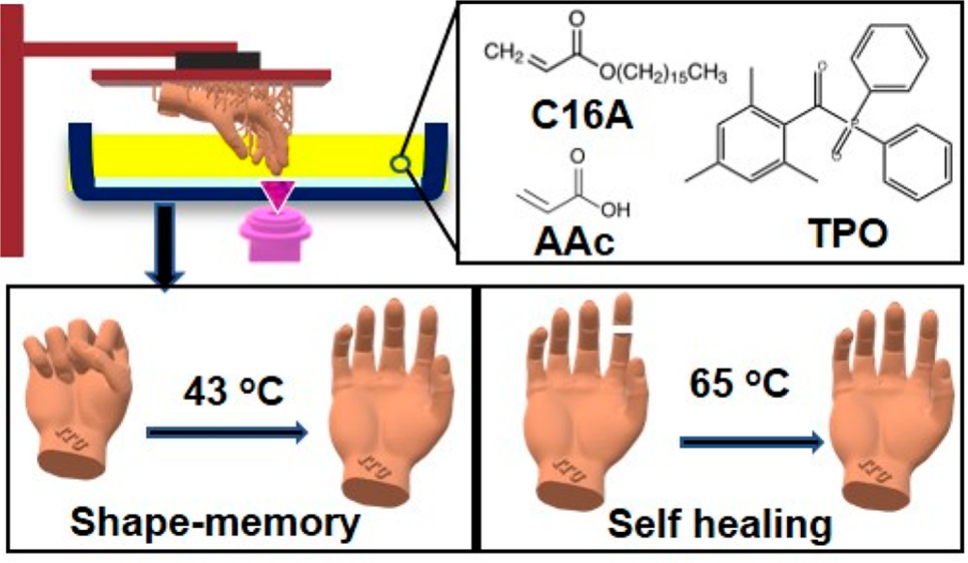

Additive manufacturing of smart materials that can be dynamically programmed with external stimuli is known as 4D printing. Among the 4D printable materials, hydrogels are the most extensively studied materials in various biomedical areas because of their hierarchical structure, similarity to native human tissues, and supreme bioactivity. However, conventional smart hydrogels suffer from poor mechanical properties, slow actuation speed, and instability of actuated shape. Herein, we present 4D-printed hydrogels based on poly(acrylic acid) that can concurrently possess shape-memory and self-healing properties. The printing of the hydrogels is achieved by solvent-free copolymerization of the hydrophilic acrylic acid (AAc) and hydrophobic hexadecyl acrylate (C16A) monomers in the presence of TPO photoinitiator using a stereolithography-based commercial resin printer followed by swelling in water. The printed hydrogels undergo a reversible strong-to-weak gel transition below and above human body temperature due to the melting and crystallization of the hydrophobic C16A domains. In this way, the shape-memory and self-healing properties of the hydrogels can be magically actuated near the body temperature by adjusting the molar ratio of the monomers. Furthermore, the printed hydrogels display a high Young’s modulus (up to ∼215 MPa) and high toughness (up to ∼7 MJ/m^3^), and their mechanical properties can be tuned from brittle to ductile by reducing the molar fraction of C16A, or the deformation speed. Overall, the developed 4D printable hydrogels have great potential for various biomedical applications.

## 1 Introduction

Additive manufacturing (AM), widely recognized as 3D printing, is an advanced technology to fabricate end-use items with innumerous complex geometric shapes according to computer-aided design.^1−3^ This disruptive technique is regarded as one of nine pillars of the fourth industrial revolution (Industry 4.0) owing to its automated production nature and mass-customized and on-demand manufacturing capacity.^1,4,5^ In the past decade, numerous prodigious advances have been made in AM, including the emergence of 4D printing.^1,6^ The concept of 4D printing was first initiated by Skylar Tibbits in 2013 by considering “time” as the fourth dimension.^7^ It is a revolutionary version of 3D printing, in which the size, shape, dimension, and physiochemical properties of printed structures can be dynamically programmed with the aid of various stimuli, such as pH, temperature, electricity, and magnet.^6,8^ 4D printing is a space-age technique that has incredible potential in numerous applications, particularly where dynamic adaptation is required, e.g., soft robotics, aerospace, and biomedical application.^9^ Nevertheless, 4D printing is still in its earliest development stage, and substantial efforts must be made for its real-world application.^10,11^ Availability and costs of printable smart materials, scalability, affordability, and simplicity of the printing technology are some of the key challenges that need to be addressed.^11,12^

Shape-memory polymers (SMP) and smart hydrogels are the two most commonly used polymeric materials for 4D printing.^13^ SMP-based 4D printing is accomplished chiefly by a series of thermomechanical programming, which consists of 3D printing of SMP, heating, fixing a temporary shape under a mechanical load, cooling, mechanical unloading, and final actuation by heat.^14,15^ Although the mechanical strength and actuation speed of SMPs are generally an order of magnitude higher than that of smart hydrogels, their applications in the biomedical field are limited due to the lack of bioactivity, rigidity, and poor permeability. Furthermore, the actuation temperature of most of SMPs is either too high (above 50 °C) or too low (below 20 °C), which is not suitable for biomedical applications.^16^

Alternatively, the 4D printing of smart hydrogels is mainly based on their swelling properties, which are influenced by several factors such as temperature, pH, the concentration of ions, and solvent type.^17^ Each of these factors acts as an external stimulus to create dynamic shape change in the hydrogel network. Smart hydrogels are the most extensively studied materials in the medical application due to their hierarchical structure and capability to simulate important biochemical characteristics of the native biological environment.^18,19^ However, inferior mechanical properties such as a low elastic modulus, and low strength, slow response rates, and instability of actuated shape are common drawbacks for hydrogel-based 4D printing (Table S1).^20^ Thus, there has been growing interest in developing printable, mechanically robust, shape-memory hydrogels that can be actuated quickly at around body temperature.

Acrylic acid (AAc)-based hydrogels are the most extensively studied materials in various medical and healthcare applications because of their supreme biocompatibility, good water adsorption capacity, and suitability for physiochemical modification.^21,22^ For instance, our previous work has demonstrated that modification of AAc-based hydrogels by crystallizable, hydrophobic n-octadecyl acrylate (C18A) monomer units dramatically improves their mechanical properties and enables them to possess temperature-programmed shape-memory behavior.^23,24^ More interestingly, the fabricated hydrogels could also repair themselves from mechanical damage with the aid of thermal stimuli, which can momentously expand their lifetime.^24,25^ Unfortunately, the actuation temperature of the hydrogels modified by C18A is considerably higher than the body temperature, which could hinder their application in the biomedical field. We recently observed that the actuation temperature for the acrylate-modified materials could be lowered by reducing the number of methylene groups in the crystalline acrylates.^26^ Consequently, n-hexadecyl acrylate (C16A), which has a smaller number of methylene groups, drew our attention. Meanwhile, we hypothesized that C16A could be mixed with AAc to form a stable liquid resin at room temperature for vat photopolymerization-based AM because of its low melting point (17 °C). Furthermore, the cytocompatibility of C16A-contained polymeric networks and their biomedical application have been reported in several studies.^27,28^

**Figure 1:**
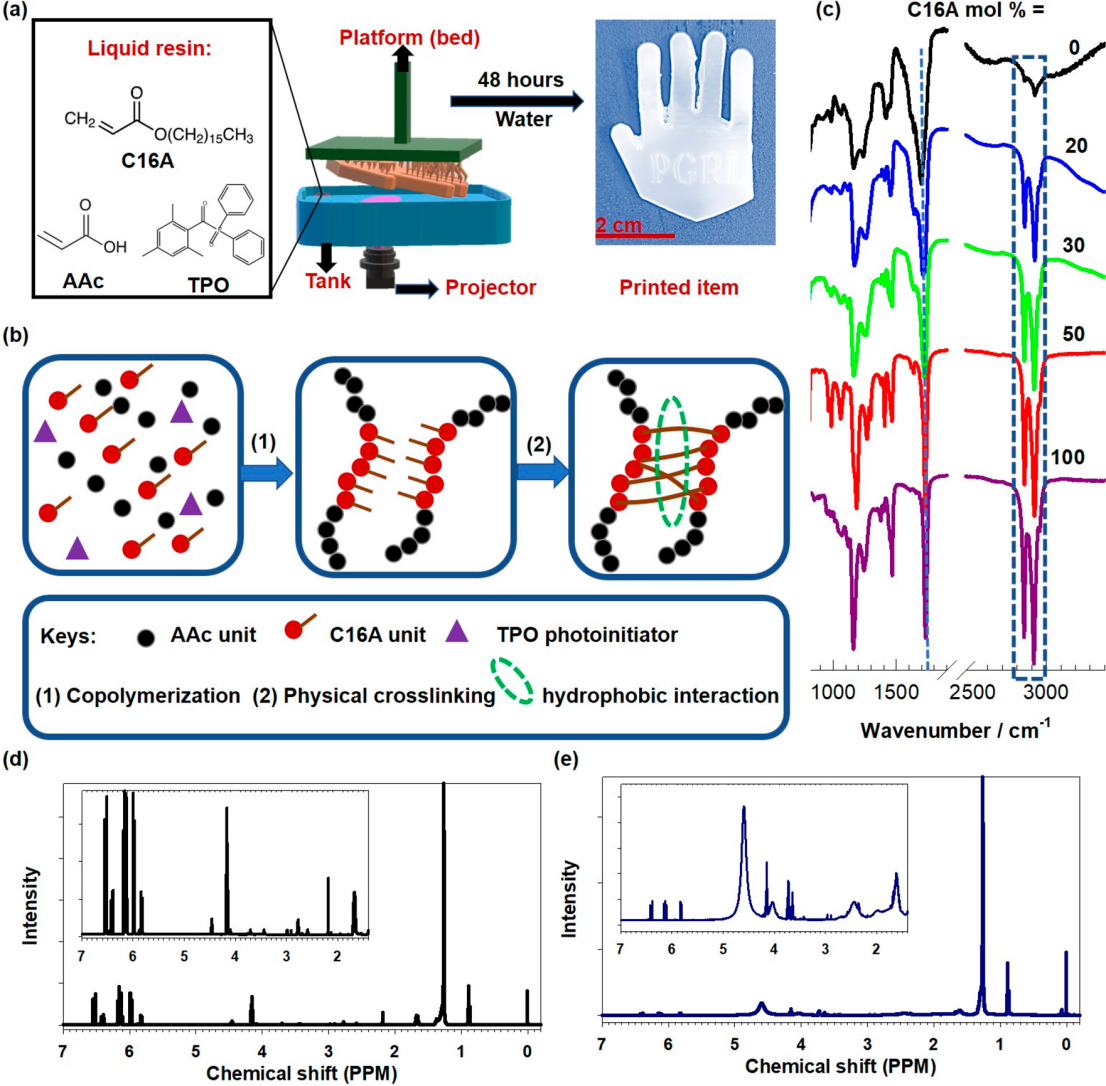
(a) Schematic illustration describing the patterned synthesis of PAAc hydrogels containing C16A units via stereolithography. (c) FTIR spectra of the printed hydrogels containing various amounts of C16A together with pure PAAc and PC16A. The dashed vertical lines indicate wavenumbers of 1730, 2854, and 2920 cm^–1^ (from left to right). (d,e) ^1^H NMR spectra of PAAc-C16A monomer mixture (C16A mol % = 30) before (d) and after printing (e).

In this work, we present 4D-printed hydrogels that can concurrently possess shape-memory and self-healing abilities near the human body temperature. The formation of the hydrogels is based on hydrophilic poly(acrylic acid) (PAAc) chains containing different molar fractions of hydrophobic C16A segments. The printing of these hydrogels can be performed in a stereolithography (SLA)-based commercial resin printer, and the physical cross-linking of the copolymer chains can be achieved via hydrophobic associations and crystalline domains of hydrophobic segments without adding a chemical cross-linker (Figure 1a).^29^ The melting and crystallization temperatures of the printed hydrogels are 38–40 °C and 25–29 °C, respectively, which allows them to possess a shape-memory effect near the human body temperature by optimizing the C16A content. This exceptional property provides us with a favorable opportunity to program 4D printing of these hydrogels near human body temperature. Besides, the printed hydrogels exhibit 23–215 MPa of Young’s modulus and up to ∼7 MJ/m^3^ of toughness together with a brittle-to-ductile transition by decreasing the C16A molar fraction or the strain rate. Furthermore, physical and structural damage in the printed hydrogels can be recovered by heating them above the melting temperature of the C16A crystalline domains.

## 2 Results and Discussion

SLA printing of PAAc-based self-healing and shape-memory physical hydrogels was achieved by solvent-free copolymerizing the hydrophilic AAc and hydrophobic C16A monomers in the presence of TPO photoinitiator followed by swelling in water (Figure 1b). The mixture of the monomers is a transparent liquid at room temperature as the melting points of both AAc and C16A are lower than room temperature (14 and 17 °C, respectively). The DSC result also confirms that the melting and crystallization temperatures of all the monomer mixtures are below room temperature (Figure S1). This is essential for the successful printing of the monomers in commercial SLA printers without providing any specific external environment.^30,31^ Moreover, any surfactants or chemical cross-linkers are not required during the polymerization and cross-linking process. This could certainly be advantageous in designing biomedical materials, since most of surfactants and chemical cross-linkers are regarded as cytotoxic.^32^ Several printing parameters, such as exposure time, light-off delay, and lifting distance, were studied to optimize the printing condition for the monomers. We found that the exposure time must be long enough to fully polymerize the monomer mixture, improve the bed adhesion ability of the print, and eventually enhance the printing quality. Therefore, bottom exposure time and exposure time for other layers were set at the maximum value the printer could provide. For instance, the factory-provided commercial resin only requires 30 s for the bottom exposure and 4 s for the normal exposure, which were extended to 70 and 10 s to print our monomer mixtures. Additionally, the printed hydrogels were further post-cured and rinsed with water and ethanol to make sure that they are completely free of any unreacted monomers.

The chemical fingerprint of the printed hydrogels was evaluated by FTIR and NMR spectroscopy. Figure 1c shows the spectra of PAAc hydrogels with various C16A contents together with their components, namely PAAc and PC16A. The characteristic stretching band of the carbonyl groups of the hydrogels, PAAc, and PC16A appears at around 1730 cm^–1^.^33,34^ The peaks at 2854 and 2920 cm^–1^ for C–H stretching of the alkyl group are characteristic for the C16A units,^35^ and their intensity increases with increasing C16A content. Furthermore, no significant shift of adsorption bands was identified for the copolymer hydrogels suggesting that the interaction between their AAc and C16A segments is physical rather than chemical.^36^ The ^1^H NMR spectra of the PAAc-C16A monomer mixture (C16A mol % = 30) before and after printing are shown in Figure 1 d and e. The chemical shift appeared at 1.3 ppm (−CH_2_– protons), 4.4 ppm, 4.0 ppm (−OCH_2_ protons), 0.9, and 3.6 ppm (−CH_3_ protons) are corresponded to the C16A segment in the hydrogel.^37^ Meanwhile, the chemical shifts were observed at 6.4 ppm (cis protons), 6.15 ppm (germinal protons), and 5.9 ppm (trans protons) for the AAc segment.^38^ In addition, several strong peaks were found between 5.8 and 6.6 ppm for the monomer mixture representing C=C double bond, and they disappeared in the printed hydrogel expectedly.^39^

**Figure 2:**
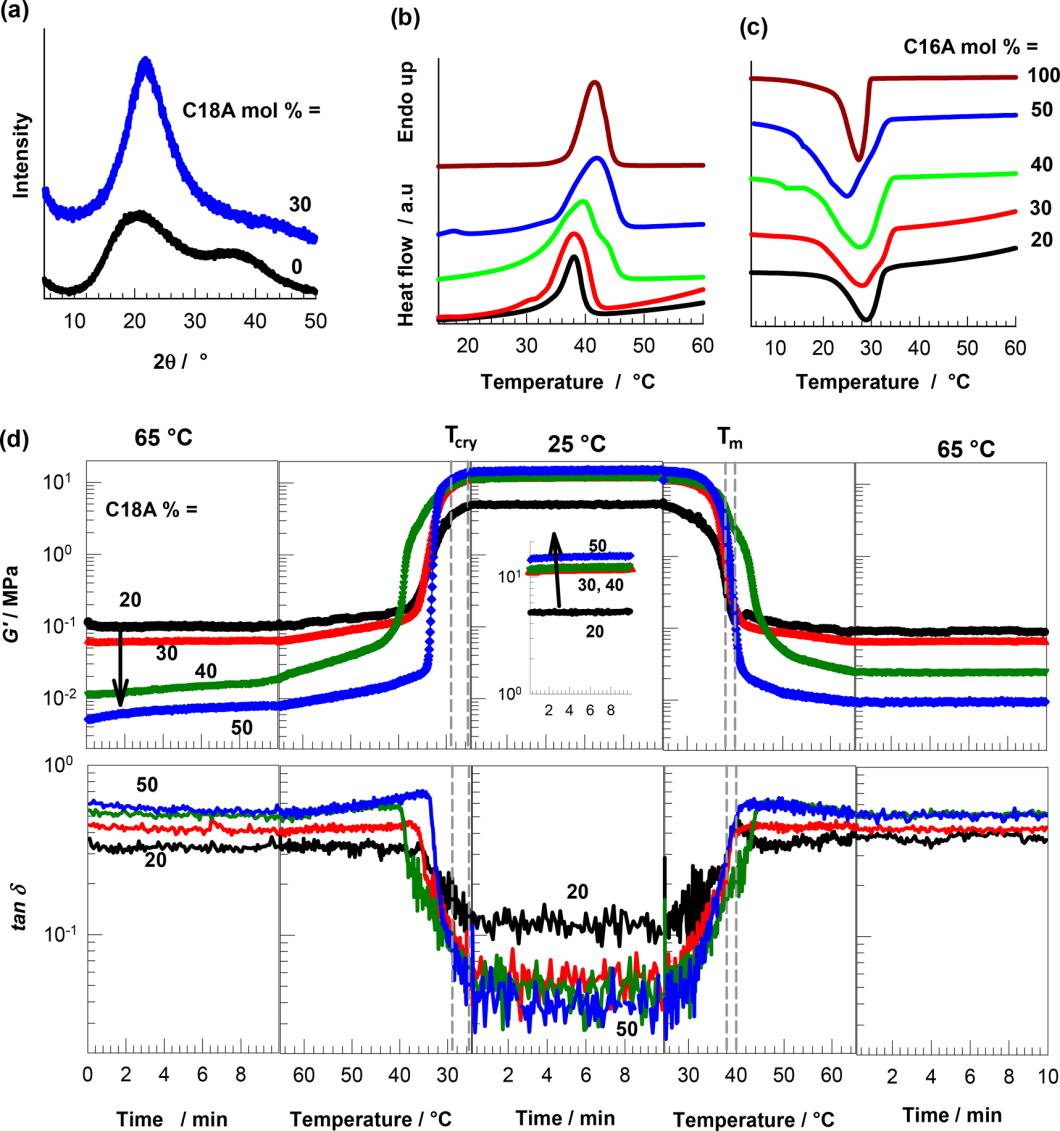
(a) XRD spectra of the printed hydrogels with 0 and 30 mol % of C16A. (b, c) DSC traces of the printed hydrogels with different molar fractions of C16A during heating (b) and cooling (c). (d) The variation of the storage modulus *G*′ and loss factor *tan δ* of PAAc hydrogels with different C16A contents during a cooling–heating cycle between 25 and 65 °C. The ranges of *T*_m_ and *T*_cry_ of the hydrogels are indicated by vertical dashed lines. Heating and cooling rates = 1 °C·min^–1^. ω = 6.28 rad·s^–1^. γ_o_ = 0.1%.

The crystalline structure of the printed PAAc-C16A hydrogels was evaluated by XRD. As shown in Figure 2a, the XRD pattern of pure PAAc exhibits a typical broad band at 2θ in between10 and 50°, indicating its amorphous nature,^40^ whereas a single sharp peak appeared at 2θ = 21.7° for the hydrogel with 30 mol % of C16A, corresponding to the hexagonal lattice of the side alkyl chains of C16A units.^2^ The temperature-dependent phase transition behavior of the swollen hydrogels due to the presence of crystalline hydrophobic C16A domains was demonstrated by DSC. All the C16A-contained hydrogels display distinct melting and crystallization peaks, as shown in Figures 2b and c, respectively. As the molar fraction of C16A is increased, the melting temperature (*T*_m_) of the hydrogels increases from 38 to 40 °C while their crystallization temperature (*T*_cry_) decreases from 29 to 25 °C (Table 1). Thus, the difference between *T*_m_ and *T*_cry_, denoted by Δ*T*, increases with increasing C16A content in the printed hydrogels. This could be resulted of the increased density of the crystalline domain in the hydrogel that requires the adsorption/release of a higher amount of thermal energy during the melting/crystallization.^41^ The degree of crystallinity (f_cry_) defined as the fraction of C16A segments forming alkyl crystals, increases from 7.5 to 15% with increasing C16A content from 20 to 50 mol %. Simultaneously, the equilibrium water content (EWC) in the printed hydrogels decreases from 51 to 7% due to the decreasing hydrophilicity of the copolymer and increasing degree of crystallinity acting as physical cross-links in the hydrogels.^23,42^

**Table 1:** Melting (*T*_m_) and Crystallization Temperatures (*T*_cry_), Δ*T* (= *T*_m_ – *T*_cry_), the Degree of Crystallinity (*f*_cry_), and Equilibrium Water Content (EWC) of the SLA Printed PAAc Hydrogels with Different Molar Fractions of C16A

C16A mol %	*T*_m_/°C	*T*_cry_/°C	Δ*T*/°C	*f*_cry_ %	EWC %
20	38.0 ± 0.2	28.6 ± 0.4	9.4	7.5 ± 0.1	51 ± 5
30	38.0 ± 0.1	27 ± 1	11	12 ± 1	24 ± 3
40	39.9 ± 0.3	27 ± 1	13	15 ± 1	16 ± 1
50	40.4 ± 0.6	25 ± 1	15	15 ± 1	7 ± 2

The effect of temperature on the storage modulus (*G′*) and loss factor (*tan δ*) of the hydrogels was investigated by oscillatory temperature-sweep tests conducted at an angular frequency ω of 6.28 rad·s^–1^ and a strain amplitude γ_o_ of 0.1%. Figure 2d shows *G′* and *tan δ* of the hydrogels with different molar fractions of C16A during a cooling–heating cycle between 25 and 65 °C. The ranges of *T*_m_ and *T*_cry_ of the hydrogels are designated by dashed vertical lines. All the printed hydrogels exhibit clear reversible changes in *G′* and *tan δ* below and above their phase transition temperatures, whose magnitude is proportional to the molar fraction of C16A in the hydrogels. At *T* < *T*_m_, the higher the C16A content, the larger the modulus due to the increasing degree of crystallinity, while at *T* > *T*_m_, the modulus decreases with increasing C16A content which we attribute to decreasing viscosity, as evidenced by the increase of *tan δ*. Thus, the most drastic changes were observed for the hydrogels with 50 mol % of C16A, in which the modulus *G′* 1750-fold changes (between 14 MPa to 8 kPa) when the temperature is varied between 25 and 65 °C. Simultaneously, the loss factor changes between 0.04 ± 0.01 and 0.5, indicating a reversible transition between strong and weak hydrogel in terms of viscoelastic behavior. Such a significant and reversible change in the modulus accompanied by a strong-to-weak gel transition in response to the temperature is important in various application areas, including soft robotics and 4D printing.^23,42^ For instance, the hydrogel could be applied to design a gripping system that capable of repeated gripping and opening through varying temperature at below or above the body temperature. More interestingly, the printed hydrogels could be also used as a drug carrier to grasp and release certain drugs by applying a triggered folding/unfolding program.

**Figure 3:**
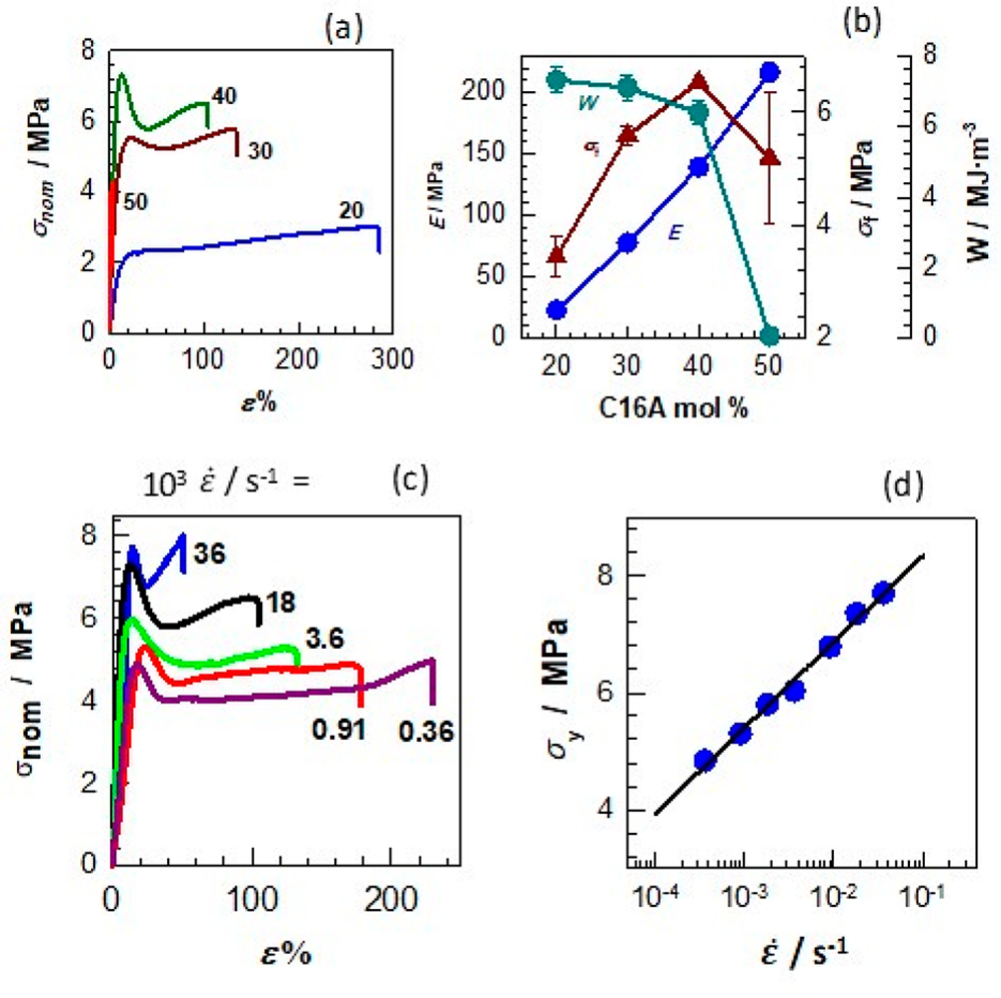
(a) Tensile stress–strain curves of the printed hydrogels containing different mol % C16A. ε̇ = 1.8 × 10^–2^ s^–1^. (b) Fracture stress σ_f_, Young’s modulus *E*, and toughness *W* of the printed hydrogels with different C16A mol %. (c) Tensile stress–strain curves of the printed hydrogels containing 40 mol % of C16A at different strain rates ε̇ as indicated. (d) Yield stress σ_*y*_ of the hydrogels plotted against the strain rate ε̇. The symbols are the experimental data, and the solid line is the best-fitting line to eq 1.

Next, we evaluated the mechanical performance of the printed hydrogels by uniaxial tensile testing at a fixed strain rate ε̇ of 1.8 × 10^–2^ s^–1^. Figure 3a shows stress–strain curves of the hydrogels with various C16A contents, as indicated. Their mechanical parameters, namely Young’s modulus *E*, fracture stress σ_f_, and toughness *W* (energy to break), are compiled in Figure 3b. The modulus drastically increases with C16A content and becomes 216 ± 8 MPa at 50 mol % C16A. Because there are no chemical cross-links in the hydrogels, the increase in the modulus with C16A content reflects the simultaneous increase in the number of crystalline domains acting as physical cross-links. Moreover, a brittle-to-ductile transition is observed for the printed hydrogels when the C16A content decreases below 50 mol %. For example, the printed hydrogel with 50 mol % C16A is extremely brittle, i.e., the elongation at break ε_f_ is only 2.9 ± 0.8% and toughness W is only 0.07 ± 0.003 MJ/m^3^, while the toughness *W* shows a 90-fold increase (6.39 ± 0.31 MJ/m^3^) when the C16A content is decreased to 40 mol %.

The brittle-to-ductile transition between 50 and 40 mol % C16A is accompanied by the appearance of a necking behavior. The hydrogel with 50 mol % C16A fractures in a brittle fashion without necking, while necking appears at lower C16A contents with yield stress increasing with C16A content up 40 mol %, leading to a significant toughness improvement. This increase in toughness can be attributed to the coexistence of crystalline domains and hydrophobic associations in the hydrogels. The brittle C16 crystals microscopically fracture at the yield point by dissipating energy, while the hydrophobic associations acting as weak cross-links keep the hydrogel sample together.^24,43,44^ This mechanism of toughness improvement is similar to the double-network hydrogels composed of ductile and brittle networks.^45^ The hydrogel with 20 mol % C16A exhibits a modulus of 23 ± 3 MPa, 7.31 ± 0.36 MJ/m^3^ of toughness and sustains 3.4 ± 0.4 MPa stresses at 281 ± 36% elongation, which verifies its soft and pliable mechanical behavior (Figures 3a, b).

Because of the physical nature of the present hydrogels, their mechanical properties depend on the time scale of the mechanical tests, as seen in Figure 3c, where the stress–strain curves of the hydrogels with 40 mol % of C16A recorded at various strain rates ε̇ are shown. The modulus *E*, yield stress σ_*y*_, and the fracture stress σ_f_ increase while the elongation ratio at break ε_f_ decreases as the strain rate ε̇ is increased. This behavior is typical for mechanically strong physical hydrogels and reflects the dissociation and association of their cross-links.^46^ Because the relaxation time of the network chains decreases with increasing strain rate ε̇, and they become more stretched at a given strain as ε̇ is increased. As a consequence, higher forces are produced on the physical cross-links facilitating their breaking at lower strains.^24,47^ Thus, the resistance to deformation represented by the modulus, yield stress, and fracture stress increase with increasing strain rate. According to the Eyring theory assuming that the polymer segments must overcome an energy barrier at the yield point, the yield stress σ_y_ is directly proportional to the logarithm of ε̇ by^48−50^
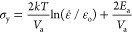
1where *V*_a_ is the activation volume, ε_o_ is the pre-exponential factor, *E*_a_ is the activation energy, and *kT* is the thermal energy. The symbols in Figure 3d represent the experimental σ_y_ vs ε̇ data in a semilogarithmic plot while the solid line is the best fitting to eq 1 yielding the activation volume (*V*_a_) as 12.6 ± 0.3 nm^3^. We should note that *V*_a_ represents the volume of the segments moving as a whole during yielding. *V*_a_ thus found is much larger than the value reported before for covalent systems,^51,52^ suggesting activation of larger units, e.g., side alkyl chain crystals.

**Figure 4:**
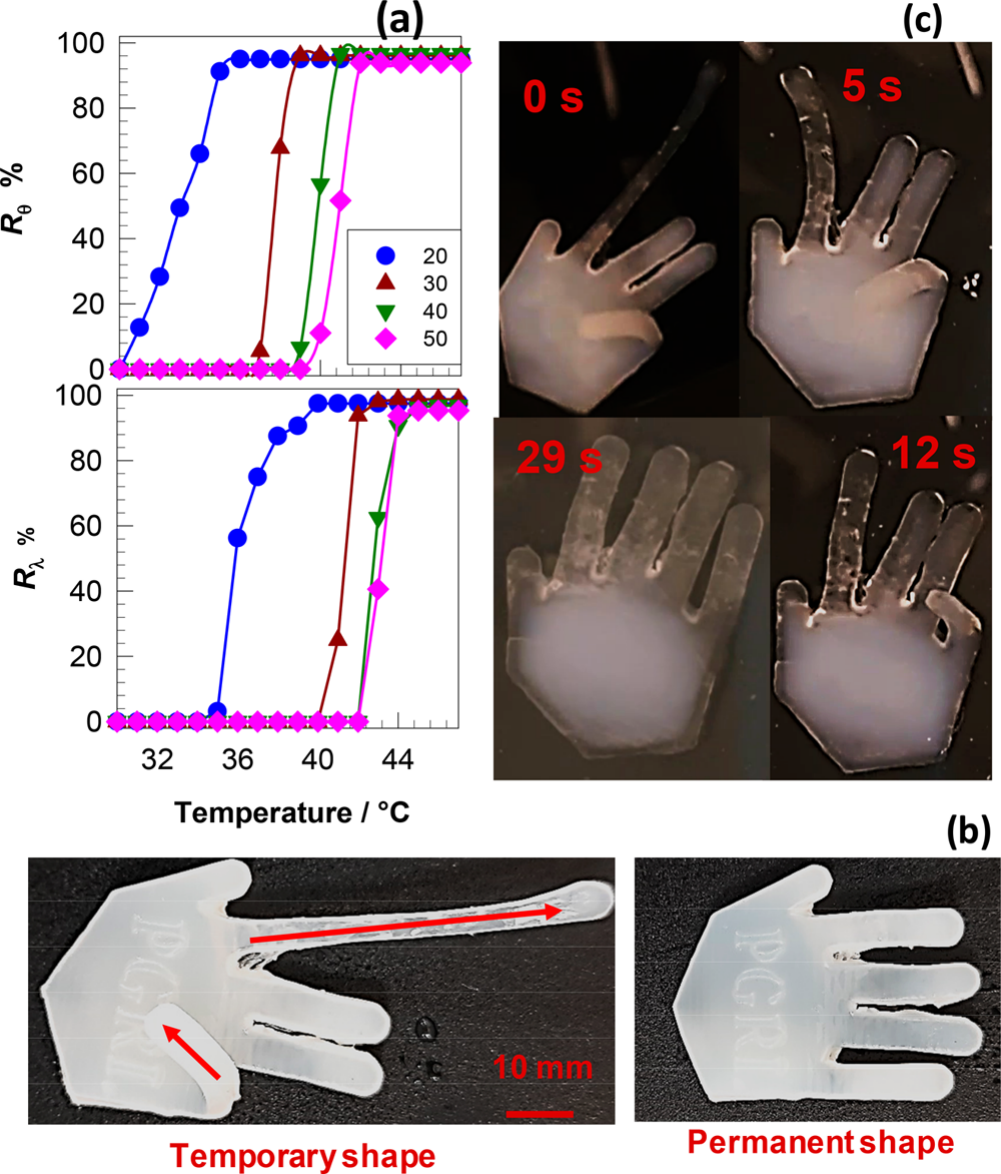
(a). The temperature-dependent shape-recovery ratios of the printed hydrogel samples after bending *R*_θ_ and stretching *R*_λ_ (b, c) A printed robotic hand in temporary and permanent shapes (b) and the shape-recovery process in water at 43 °C (c).

The shape-memory property of the hydrogels was studied by observing their ability to return to their original shape as a response to temperature after bending and stretching. The shape-recovery ratios *R*_θ_ and *R*_λ_ of the printed hydrogel specimens after bending and stretching are shown in Figure 4a as a function of temperature. The general trend is that the actuation temperature for recovering the original shape from stretching is generally 2–3 °C higher than that from bending. This could be related to the smaller dimension of the deforming area and smaller deformation ratio in the case of bending compared to stretching.^53,54^ Moreover, the ability to fix the temporary shape below *T*_m_ increases with the increasing C16A content of the hydrogels. For instance, the hydrogel with 20 mol % C16A is unable to fix its temporary bent shape even at 30 °C due to its low degree of crystallinity, while the hydrogels with 40 and 50 mol % C16A fix their temporary shapes up to 39 °C. Moreover, the printed hydrogels, except those with 20% of C16A, could recover back to their permanent shape over a narrow temperature range nearby the human body temperature, e.g., 37–42 °C.

To demonstrate the 4D printing effect of the hydrogels, a robotic hand of 48 mm in length, 36 mm in width, and 5 mm in thickness was printed by SLA. Then a temporary shape for the hand was created at 43 °C by folding one of the fingers and stretching another one prior to cooling it to 20 °C (Figure 4b). Afterward, the hand was placed in water at 43 °C to realize the shape recovery (Figure 4c and Movie S1). The result shows that the shape recovery from both folding and stretching occurs simultaneously, and the printed hand can fully return to its original shape in less than 1 min.

**Figure 5:**
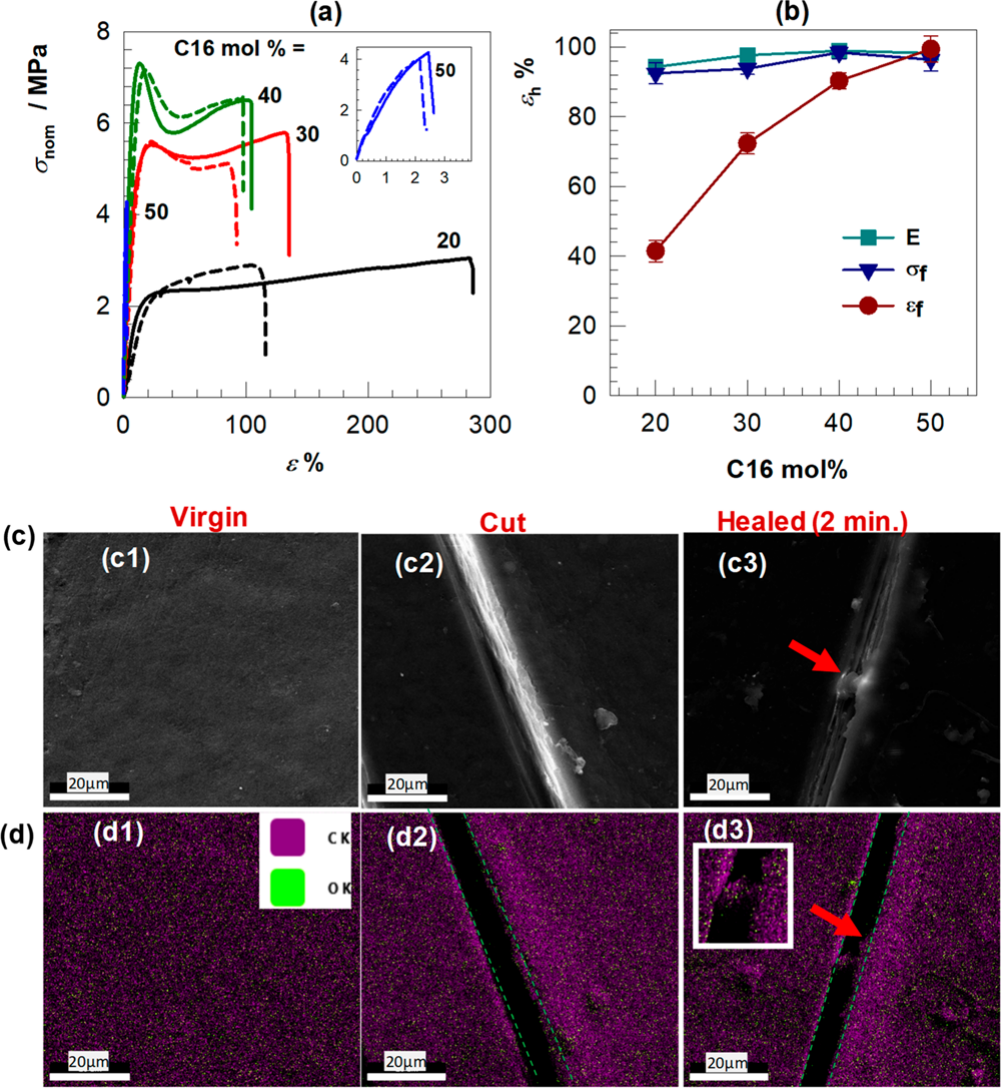
(a) Tensile stress–strain curves of the virgin hydrogels (solid line) and healed hydrogels (dashed line) containing the different molar fractions of C16A. (b) The healing efficiency ε_h_ of the printed hydrogels in respect of fracture stress σ_f_, the modulus *E*, and fracture strain ε_f_. (c, d) SEM images (c) and the corresponding elemental mapping (d) of the hydrogels to provide evidence for initiation of the healing process. The images were taken from virgin (c1,d1), damaged (c2, d2), and healed samples (c3, d3). The green line in the elemental mapping images indicates the scratch area. Ck and Ok in d1 represents carbon and oxygen element, respectively.

Finally, we assessed the self-healing performance of the printed hydrogels by comparing their mechanical performance in their virgin and healed states. The healing in the hydrogels was tested by first cutting the specimens in the middle, followed by keeping the cut surfaces together at 65 °C for 1 day, and finally cooling to room temperature. The strain–stress curves for the virgin hydrogels (solid line) and healed hydrogels (dashed line) with different molar fractions of C16A are depicted in Figure 5a, and the healing efficiencies with reference to fracture strength σ_f_, Young’s modulus *E*, and fracture strain ε_f_ are compiled in Figure 5b. It is seen that the shape of the stress–strain curves, including the yield point, could be recovered after 1 day of healing at 65 °C revealing the recovery of the original microstructure. The healing efficiencies with respect to *E* and σ_f_ are found to be above 90% regardless of the C16A content in the hydrogels. In contrast, the healing efficiency with regard to ε_f_ rises from approximately 40 to 100% with increasing C16A content from 20 to 50 wt %. The relatively low healing efficiency in respect of the fracture strain could be attributed to the incomplete overlap of the cut surfaces of damaged hydrogels. This result also implies that the dominance of the C16A hydrophobic domains is critically important for the healing process since the healing process is accomplished by the reassociation of hexadecyl side chains above the melting temperature of C16A segments.^55,56^

We further examined the initiation of the self-healing process by SEM-EDX measurements. For this purpose, the healing temperature and the healing time were reduced from 65 to 43 °C and for 1 day to 2 min, respectively, to slow down the healing process. A linear crack was first created on a printed hydrogel specimen with 40 mol % C16A by a razor blade, and then SEM images from the crack region were taken before and after healing at 43 °C for 2 min. Figure 5c shows SEM images of a hydrogel sample before cutting (c1), after cutting (c2), and after 2 min of healing (c3). It is seen that although the healing time is only 2 min, the thickness of the cut starts to decrease, and a bridge appears between the cut surfaces, as indicated by a red arrow. Bridging of the cut surfaces is also seen in Figure 5d1–d3, presenting EDX maps of the spatial distributions of oxygen (green) and carbon atoms (pink) in the cut region. Because AAc and C16A units of the hydrogels have the formula C_3_H_4_O_2_ and C_19_H_36_O_2_, respectively, the spatial density of carbon in the hydrogel is much higher compared to that of oxygen (Table S2), which is also seen in EDX maps with a dominated pink color in which green regions are distributed (Figure S2). As highlighted in the inset to Figure 5d3, the polymer region connecting the cut surfaces consists of both carbon and oxygen atoms indicating that the homogeneous dispersion of the AAc and C16A segments is not disturbed during the physical damage and healing of the hydrogel. The results also reveal that the healing process for the hydrogels can be initiated at a temperature near to the human body temperature, although it would take a considerably long time to heal a wholly separated part in this case.^57^

## 4 Conclusion

In summary, we successfully printed a series of shape-memory, self-healing, and mechanically robust hydrogels by copolymerizing AAc and C16A monomer mixtures in the presence of a photoinitiator via the SLA technique using a commercial resin printer. The printed hydrogels were further placed in water to facilitate physical cross-linking via hydrophobic interactions between the hexadecyl side chains of C16A. The hydrogels exhibit a reversible strong-to-weak gel transition at 36–41 °C due to the presence of the C16A crystalline domains. The magnitude of the transition is proportional to the molar fraction of C16A in the hydrogels. The mechanical properties of the printed hydrogels can be altered from brittle to ductile behavior by decreasing the C16A content or the strain rate. For example, both the toughness and elongation at the break increase by 90-fold when the C16A content is decreased from 50 mol % to 40 mol %. At a fixed strain rate, the hydrogel with 40 mol % C16A shows a distinct necking behavior and has the highest fracture strength among the studied hydrogels. The shape-memory and self-healing properties of the hydrogels can be actuated near the human body temperature, while higher temperature encourages faster healing of the hydrogels. Particularly, the shape-memory effect for the hydrogels with 40 mol % C16A can be triggered just above human body temperature (39–43 °C). The hydrogels with a higher amount of C16A generally show better shape-memory and self-healing performances. Overall, AAc resin containing 40 mol % C16A provides the optimum condition to produce 4D printable, mechanically robust, shape-memory, and self-healing hydrogels that hold great potential for various biomedical applications.

## 3 Experimental Section

### 3.1 Preparation of AAc-C16A Resins and SLA Printing

Diphenyl(2,4,6-trimethylbenzoyl) phosphine oxide (TPO, Sigma-Aldrich) and n-hexadecyl acrylate (C16A, TCI Chemicals) were used without any treatments. Acrylic acid (AAc, Merck) was passed through an alumina column (Sigma-Aldrich) to remove its inhibitor. The AAc-C16A resins with 20–50 mol % of C16A were prepared by melting the C16A monomer at 35 oC on a magnetic stirrer and mixing it with the AAc monomer. After the addition of 2 wt % TPO as a photoinitiator (with respect to total monomers) and cooling the solution down to room temperature (23 ± 2 °C), printing was performed with a Halot-one SLA printer (Creality, China). The minimum resolution of the printer is 50 μm in X and Y directions, and 10 μm in Z direction. We used the default setting to print out our monomer mixture, in which 50 μm of resolution was applied in all the three directions. The bottom exposure time was set at 70 s, while for other layers, it was set at 10 s. After printing each layer, the printed part was allowed to cool down for 5s before starting to print the next layer. The printed samples were then cured under 405 nm laser light for 10 min and rinsed with ethanol for 4–5 times to remove unreacted monomers. Finally, the samples were immersed in a large amount of water for 2 days to facilitate physical cross-linking.

### 3.2 Characterization

Fourier-transform infrared spectra (FTIR) of the printed hydrogels were obtained using a Carry 630 FTIR spectrometer (Agilent Technologies). The samples were placed on top of an ATR accessory, and the spectra were recorded in the wavenumber range of 4000–400 cm^–1^. ^1^H NMR spectra of the printed hydrogels were obtained by VNMRS 500 MHz spectrometer (Agilent Technologies, CA, USA). For the measurements, 10 mg of sample was dissolved in 0.75 mL of deuterated chloroform (CDCl_3_), then the solution was placed in 4 mm NMR tubes. XRD measurement was performed on a Panalytical Empyrean instrument (Malvern Panalytical Ltd., Malvern, U.K.) attached with Cu-Ka radiation. The data were collected in the angular range 2θ of 1–50° with a scan rate of 1°·min^–1^.

Differential scanning calorimetry (DSC) measurements for the liquid resins and the printed hydrogels were conducted on a Diamond differential scanning calorimeter (PerkinElmer, Massachusetts, United States). Prior to recording the DSC scans of the resins, the liquid resins were solidified by placing them in a refrigerator at −20 °C for one h. Afterward, 10 mg resin was loaded into an aluminum crucible of the instrument, and the sample temperature was first raised from −20 to 40 °C at 5 °C/min, then reduced to −20 °C at the same rate. In the case of the printed hydrogels, the sample temperature was first raised from 0 to 80 °C at 5 °C/min and then reduced to 0 °C at the same rate. During the measurements, nitrogen gas (a feed rate of 19.8 mL. min^–1^) was swept over the cell to prevent the samples from reacting with the environment. The degree of crystallinity (*f*_cry_) for the hydrogels was calculated by
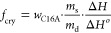
2Where *m*_s_ and *m*_d_ are the weights of the swollen and dry specimen, respectively, *w*_C16A_ is the weight fraction of C16A in the copolymer, Δ*H* is the melting enthalpy of the specimen, and Δ*H*_0_ is the melting enthalpy of crystalline C16A units, taken as 149 J/g.^34^

The rheological behavior of the printed hydrogels as a response to temperature was evaluated by temperature-sweep oscillation tests on a Bohlin Gemini 150 rheometer (Bohlin Instruments, UK). A disk-shaped sample with a diameter of 20 mm and a thickness of 1 mm was firmly fixed between the parallel plate of the system. A cooling–heating cycle in the range of 25–65 °C was then applied to the sample at a fixed angular frequency (ω) of 6.3 rad/s and strain amplitude (γ_o_) for 0.1%. The mechanical performance of the printed hydrogels was assessed by uniaxial tensile testing on a Zwick Roell Z0.5 TH universal testing machine with a 500 N load cell. The dog-bone-shaped specimen that was printed according to the standard of the International Organization for Standardization (ISO 527) was stretched at various strain rates. The stress–strain data of the printed hydrogels were recorded using a testXpert III Testing Software of Zwick/Roell instrument as the nominal stress (σ_nom_) and strain (ε). The linear part of the stress–strain curves between 0.5 and 1% strain was selected to determine Young’s modulus. The toughness *W* (energy to break) was calculated as the areas beneath the stress–strain curves until the breakpoint and the yield stress σ_*y*_ was determined from the nominal stress at the yield point. The equilibrium water content (EWC) of the printed hydrogels was determined by keeping the hydrogel specimen in water until obtaining the swelling equilibrium, which required about 2 weeks. EWC was calculated by
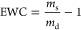
3

### 3.3 Evaluation of Shape Memory Effect and Self-Healing Performance

The shape-memory performance of the hydrogels was examined by monitoring their ability to return their original shape after stretching or bending. For the stretching test, the specimens (ISO 527) were first stretched twice their initial length (λ_o_) at 42 °C, and then the specimen was immediately placed in 25 °C of water under force to fix the elongated shape. For the bending test, flat rectangular samples (30 × 20 × 1 mm) were completely folded at 42 °C, and then the specimen was immediately placed in 25 °C of water under force to fix the folded shape. For the shape-recovery of the stretched or bent samples, they were heated up to 50 °C at a heating rate of 1–2 °C/min, during which the sample length (λ_d_) or bending angle (θ_d_) was monitored using a digital camera. Afterward, the recovery ratios followed by stretching (*R*_λ_), and bending (*R*_θ_) were determined by,
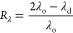
4
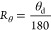
5The self-healing performance of the printed hydrogels was assessed by mechanical testing. For this purpose, the specimens were first cut from the middle to split into two pieces. Then the cut surfaces of these two pieces were kept in contact at 65 °C for 1 day to facilitate the self-healing process. After cooling to room temperature, the uniaxial tensile testing was performed on the healed specimens, and the obtained results were compared with the virgin ones. Additionally, the initial healing process for the printed hydrogel at 43 °C was monitored by scanning electron microscopy-energy dispersive X-ray spectroscopy (SEM–EDX) mapping that was performed on an FEI Quanta FEG 200 microscope (UMass Chan, MA, USA).

## Supporting Information Available

Supplementary characterization data, including SEM-EDX and DSC results (PDF)
A movie demonstrating 4D printing using P(AAc-C16A) hydrogels with 40 mol % C16A (MP4)
