# Supplementary material for: 4D Printing of Body Temperature-Responsive Hydrogels Based on Poly(acrylic acid) with Shape-Memory and Self-Healing Abilities
Source: ACS Appl Bio Mater. 2023 Jan 26;6(2):703–11. doi: 10.1021/acsabm.2c00939 (PMC9945108; doi:10.1021/acsabm.2c00939)
Supplement: Supplementary file 1 — mt2c00939_si_001.pdf [file mt2c00939_si_001.pdf]

Supporting Information

4D printing of body temperature-responsive hydrogels based on polyacrylic acid with shape-memory and self-healing abilities

*Turdimuhammad Abdullah *, Oguz Okay **

Department of Chemistry, Istanbul Technical University, 34469 Maslak, Istanbul, Turkey.

* Corresponding authors: okayo@itu.edu.tr; abudulat@itu.edu.tr

Table S1. Mechanical properties of some Polyacrylic acid (PAA)-based hydrogels.

Hydrogel composition	Test type	Elastic modulus	Fracture stress	Fracture strain	Reference
PAA	Compression	207 kPa	158 kPa	45%	[1]
PAA-PVA	Compression	71 kPa	222 kPa	69%	[1]
PAA-MBA	Tensile test	12 kPa	127 kPa	1251%	[2]
PAA-Si	Tensile test	53 kPa	281 kPa	1750 %	[2]
PAA-NCC	Tensile test	170 kPa	144 kPa	244 %	[3]
PAA-DANC	Tensile test	170 kPa	124 kPa	220 %	[3]
PAA-DCNC	Tensile test	619 kPa	300 kPa	522 %	[3]

PVA: polyvinyl alcohol; MBA: N,N′-methylene bisacrylamide; Si: silica nanoparticles; NCC: nanocrystalline cellulose; DANC: dialdehyde nanocrystalline cellulose;DCNC: dicarboxylated nanocrystalline cellulose

Table S2. Elemental composition of the virgin, cut, and healed P(AAc-C16A) hydrogels containing 40mol% of C16A confirmed by EDX.

Element		Virgin	Cut	Healed
O	Atomic.%	8.5	8.1	8.6
	Weight.%	11.5	10.4	11.1
C	Atomic.%	91.5	91.9	91.4
	Weight.%	88.5	89.6	88.8

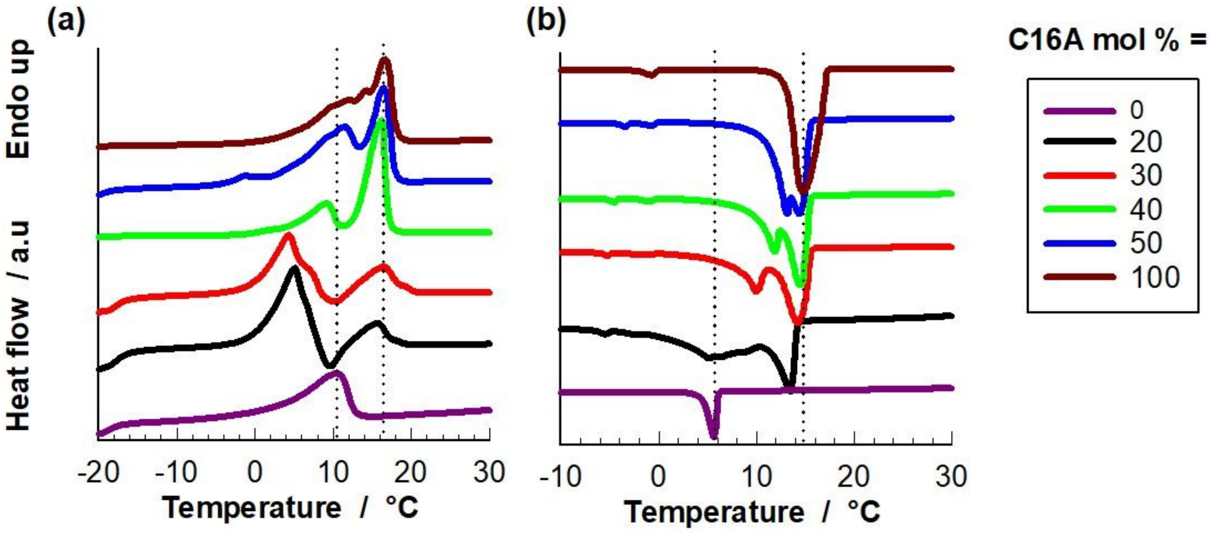

Figure S1. The DSC traces of the PAAc-C16A resin with different molar fractions of C16A during heating (a) and cooling (b)

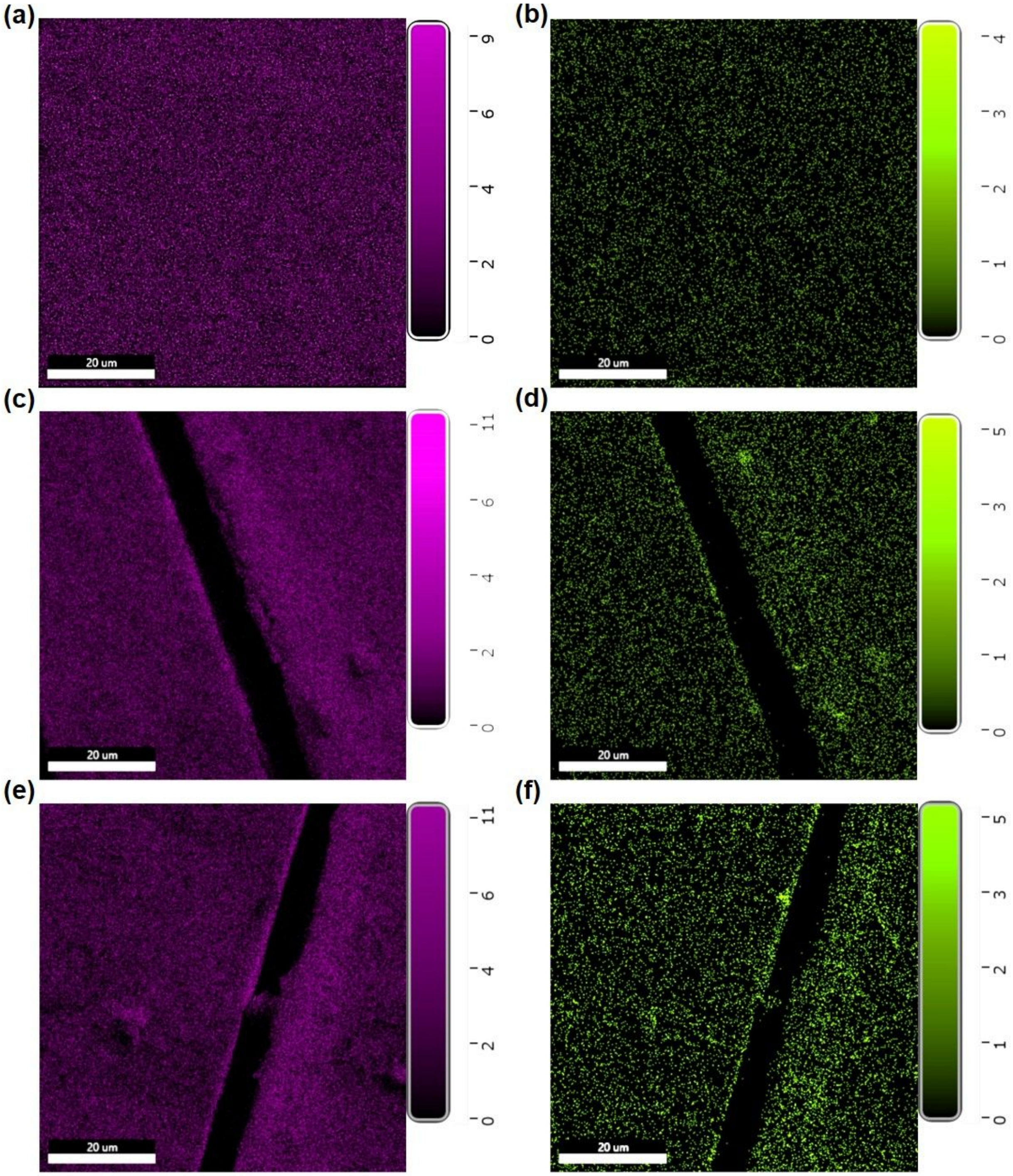

Figure S2. The spatial distributions of carbon and oxygen in the virgin (a, b), cut (c, d), and healed (e, f) hydrogel based on the SEM micrograph in Figure 5c.

References

(1) Dmitriev, I. Y.; Ivanov, V.; Bobrova, N.; Zoolshoev, Z.; Kuryndin, I.; Elyashevich, G. Hybrid Hydrogels Based on Cross-Linked Polyacrylic Acid and Polyvinyl Alcohol as Electrically Controlled Artificial Muscles. *Russ. J. Appl. Chem.* **2016**, *89* (11), 1838-1845.

(2) Sujan, M. I.; Sarkar, S. D.; Sultana, S.; Bushra, L.; Tareq, R.; Roy, C. K.; Azam, M. S. Bi-Functional Silica Nanoparticles for Simultaneous Enhancement of Mechanical Strength and Swelling Capacity of Hydrogels. *RSC Adv.* **2020**, *10* (11), 6213-6222.

(3) Hossain, M. A.; Roy, C. K.; Sarkar, S. D.; Roy, H.; Howlader, A. H.; Firoz, S. H. Improvement of the Strength of Poly (Acrylic Acid) Hydrogels by the Incorporation of Functionally Modified Nanocrystalline Cellulose. *Materials Advances* **2020**, *1* (6), 2107-2116.
